# Comparing the Achievable Sensitivity Limits of Synchrotron-based X-ray Fluorescence Imaging versus conventional X-ray absorption imaging and comparing absorbed dose levels including PET/SPECT

**DOI:** 10.1016/j.zemedi.2025.04.001

**Published:** 2025-04-25

**Authors:** Florian Grüner, Jan Scheunemann, Christoph Hoeschen, Thorsten Frenzel, Theresa Staufer

**Affiliations:** a University of Hamburg, physics department, and Center for Free-Electron Laser Science (CFEL), Luruper Chaussee 149, 22761 Hamburg, Germany; b Otto-von-Guericke University, Institute for medical technology, Faculty for electrical engineering and information technology, Magdeburg, Germany; c Department of Radiotherapy and Radiation Oncology, University Cancer Center Hamburg, Hubertus Wald Tumorzentrum, University Medical Center Hamburg-Eppendorf, Martinistrasse 52, 20246 Hamburg, Germany; d Mildred-Scheel-Cancer Career Center Hamburg, University Cancer Center Hamburg, Hubertus Wald Tumorzentrum, University Medical Center Hamburg-Eppendorf, Martinistrasse 52, 20246 Hamburg, Germany

**Keywords:** X-ray fluorescence imaging, preclinical imaging, cell tracking, pharmacokinetics

## Abstract

The field of X-ray Fluorescence Imaging (XFI) is relatively new, with recent breakthroughs in preclinical applications using synchrotron radiation, whereas X-ray tube-based absorption imaging, such as CT, is a very well-known and widely used imaging technique. Thus, the question arises how XFI and conventional X-ray imaging compare, in particular in terms of achievable detection sensitivity. In this article we briefly summarize the state of the art of XFI with the special focus on shedding light onto the reasons for why XFI has an intrinsically higher sensitivity than any other form of X-ray based absorption imaging. Since the issue of applied radiation dose limits needs to be taken into account as well, we kept the absorbed dose levels, when comparing XFI with absorption imaging, the same, but will also compare the XFI-required dose level with the levels in PET/SPECT to also allow for a quantitative comparison with these ultra-high sensitivity nuclear imaging modalities.

## Introduction

The applicability of X-ray Fluorescence Imaging (XFI) for biomedical imaging has been discussed since the mid 1980s [1]. The main use of in-vivo or in-situ XFI is the tracking of entities like functionalized gold or other nanoparticles [2,3], immune cells [4], medical drug compounds [5], small molecules [6], nano-/micro-plastic [7], antibodies and maybe even virus particles. However, in order to make such entities detectable, for instance through an in-vivo scan, they need to be labelled with XFI-markers. In case of small molecules and medical drug compounds this is typically 1-3 iodine atoms incorporated into the chemical structure of the molecules under study. For immune cells, either nanoparticles or molecular contrast agents can be used, with which the cells are incubated. Gold nanoparticles are already an XFI-marker by themselves, this is also true for many other nanoparticles as long as the marker element is heavier than zirconium for mouse-sized objects. The reason for this condition lies in the fact that XFI is based upon the excitation of typically K-shell fluorescence and in order to detect the emitted X-ray photons, their transmission through the object must be large enough, hence the XFI-marker elements must provide K-shell fluorescence lines with sufficient photon energy, typically up from 20 keV, with incident energies around 50-60 keV. This is in contrast to using lower energy X-ray photons to examine natural elemental composition ex-situ in thin samples where transmission is not a limiting factor.

XFI can be realized by either scanning the objects with pencil X-ray beams or even with cone beams as in bench-top solutions [8], which is then often referred to as X-ray Fluorescence Computer Tomography (XFCT). In this work, we fully concentrate on just the first case and here even only on synchrotron-based XFI. The reason is the following: synchrotrons, like PETRA III at DESY (Hamburg, Germany), deliver pencil beams with a flux and narrow bandwidth which is technically impossible to reach with today’s bench-top systems. As we have shown in [4], synchrotron-based XFI reaches detection sensitivity levels that even allow the tracking of labelled immune cells in living mice.

In contrast to XFI, the main application of conventional X-ray imaging is gaining anatomical data. However, also conventional X-ray imaging can be used for localizing markers such as gold nanoparticles [8]. While such works have shown interesting results, their detection sensitivity is not sufficient for many interesting challenges in biomedical imaging. In ref. 8 a very illustrative comparison between XFCT and CT is shown, where CT is not able to detect gold nanoparticles administered into a tumor-bearing mouse, while for XFCT the signals were very strong. In contrast to bench-top devices mainly used in XFCT, a synchrotron X-ray beam provides measured X-ray spectra with a drastically reduced spectral background [4], hence providing a significantly improved detection sensitivity.

In this paper we will study some very basic setups that help in understanding why the sensitivity of synchrotron-based XFI is, when using the same radiation exposure, superior to conventional X-ray imaging, including various radiography scenarios and CT.

The most fundamental physical difference between XFI and conventional X-ray imaging lies in the fact that in the latter case the contrast measured within a recorded X-ray image relies on the differences between neighboured transmitted X-ray beams’ intensities. If such intensity differences are too little with respect to the statistical noise level, then the contrast is non-detectable, the main reason for why e.g. too small tumours cannot be detected with conventional X-ray imaging. XFI on the other hand is indeed also based upon measuring a difference, however, this is not the contrast between neighboured intensities in an X-ray image, but the difference between the X-ray fluorescence lines in the measured spectrum compared to their underlying spectral background. As long as these lines show a sufficiently high statistical significance above background noise (typically 3 or 5 standard deviations), the XFI-signals are registered.

While the sensitivity of XFI is superior to that of conventional X-ray imaging, be it radiography or CT, XFI is not a background-free modality as the measured X-ray spectra entail the background from multiple Compton-scattered photons as each such Compton-scattering lowers the energy of the incident photon and can reach the XFI-lines in the spectra. In contrast to XFI, PET and SPECT are indeed background-free and thus show the highest achievable detection sensitivities in terms of minimal local amounts of the used tracers, which for PET and SPECT are radioactive, while for XFI they are non-radioactive. However, XFI offers some key advantages over PET/SPECT: (i) XFI has a higher spatial resolution [9], (ii) it offers an easy way for multi-tracking [4], (iii) XFI allows multi-scale imaging, that is, after a full-body scan single cells from biopsy can be scanned using a synchrotron beam focused down to sizes of sub-cellular scales, and, last, but not least, (iv) it does not require a radiochemistry laboratory.

Furthermore, since XFI-markers are non-radioactive, there is no physical decay of the XFI-signal, which allows for longitudinal measurements, enabling the assessment of the same specimen over time to observe the progression of marker distribution.

## Materials and Methods

### Comparative Study between XFI and X-ray Absorption Imaging

As outlined in the introduction, the types of contrast measured in XFI and conventional X-ray imaging are quite different. Here, we will analyse them in detail with the help of a very simple model, starting with radiography.

### Setup and Models

As we focus on preclinical imaging, the object in this model consists of a water sphere with a diameter of 30 mm (=”object sphere”), resembling a simplified mouse-like object. The shape of a sphere was chosen because the emission of X-ray fluorescence is isotropic, making analytical estimates quite straightforward.

To estimate the sensitivity limits, a small target sphere with a diameter of 1 mm is placed at the centre of the object sphere. This target consists of an iodine-water solution with a concentration as given by the aforementioned immune-cell tracking XFI experiment, that is, only 200 ng within one single cubic voxel of 1mm size, with all other voxels remaining iodine-free: this iodine amount was taken from a typical scan position in our in-vivo immune cell tracking work [4], but refers to the iodine mass within the entire beam volume, however, for simplifying the calculations we put this amount into only one voxel. For XFI and radiography this does not make any significant change in the signal strengths as the object is relatively small compared to the mean free path length of the incident photons. Thus, putting the entire iodine mass into just one voxel at the centre is very similar to an averaging over all voxels along the incident beam volume.

It is important to keep in mind that XFI is not affected by inhomogeneities, because one only needs to estimate the mean transmission of the fluorescence photons through an inhomogeneous object. We have seen in pre-studies that the measured XFI-spectra are very similar no matter whether a living or dead mouse, or even a mouse phantom is scanned. In other words, there is no need to measure two images for subtraction, as is required in conventional X-ray imaging when comparing the case before and after administering the contrast agent. For XFI, it is sufficient to only measure once whether there is a signal from the scanning beam volume or not.

For radiography with a contrast agent, we will refer to the process of comparing the two different images (before and after injection of the contrast agent) as “subtraction imaging”, in contrast to variations observed within a single image, which we will denote as “single imaging.” In this context, XFI is also treated as a single imaging.

Subtraction imaging involves calculating the difference between two images to eliminate common structures and enhance the visibility of specific features, such as the contrast agent, which in XFI is called the marker. By subtracting the baseline image from the image containing the marker, we can better isolate and identify the true location and distribution of the marker, reducing the likelihood of confusion with anatomical structures which poses the key challenge to X-ray absorption imaging.

As can be expected and is shown below, the single X-ray absorption imaging requires a higher amount of a marker to clearly differentiate between the tissue containing the marker and adjacent structures, such as bone.

In order to also treat more realistic objects in our numerical study, we will also generate a random inhomogeneous model, that is, a voxelated sphere which was randomly filled with soft tissue (91.2%) and bone tissue (8.8%) with 1 g/cm^3^ density for soft tissue and 1.85 g/cm^3^ for bones. The material composition was based on the International Commission on Radiological Protection (ICRP)values for soft tissue and cortical bone. The amount of bone tissue was chosen to be at the lower end and was calculated based on the fraction of bone to tissue volume for mammals (BV/TV = 10-25%) from ref. [10].

In the subtraction imaging of the inhomogeneous case, we use, as a conservative estimate, the same randomly generated voxels twice and only add the marker. It would be more realistic to generate a second inhomogeneous sphere as a living mouse, in particular, exhibits changes from one imaging scan to the next, such as those caused by breathing and motion artefacts as well as from variations in placing a mouse twice in the same imaging system before and after marker injection. However, here we want to compare XFI with the simplest radiography subtracting imaging, that is, mimicking a perfect repetition of the scan after contrast agent injection. This subtraction of two images from one another can yield significant results if the amount of marker is high enough to differentiate between the setting without and the one with the marker, hence, this difference must be larger than the statistical noise of both images. Note that subtraction imaging means doubling the absorbed dose from a single imaging.

Because each voxel has a width of 1mm there is no central voxel in a sphere of even diameter. Therefore, the marker was placed 0.5 mm off-centre in x, y and z direction.

In subtraction imaging we will calculate the significance Z in units of standard deviations for the intensities of two images $I_{1}$ and $I_{2}$ this way:

$$Z=\frac{|I_{1}-I_{2}|}{\sqrt{I_{1}+I_{2}}},$$

However, when studying only the single radiography imaging case, the data was sourced directly from a CT scan of a mouse, accounting for a broader variety of tissues, and no Monte-Carlo code was used in the case of single imaging. This has two reasons: GEometry ANd Tracking (Geant4) calculates the attenuation for each voxel based on the density and material composition at that position and randomly samples photon-matter interactions such as photo absorption, X-ray fluorescence, and Compton scattering. In contrast, a CT scan provides direct attenuation measurements, which makes it more complex and error-prone to decompose these measurements into distinct materials and densities and then recombining them using Geant4 to calculate the absorption coefficient of a voxel. Therefore, using the CT-derived attenuation values directly for analytical calculations is more straightforward.Secondly the simulations will show that the effect of scattering is negligible therefore the analytical estimates suffice in the single image case.

The 8.8% of bone (used in the first model) were verified in the creation of the second model for single imaging where a 100 µm resolution CT was contoured to get the attenuation distribution of all the organs in a mouse. As resolution we understand here the voxel size and equal to that the voxel separation. In the contoured mice CT the amount of bone voxels was about 10.4% of the whole mouse volume. So, our first estimate that about 10% of a mouse can be assumed to be bone remains true and most likely within the biological distribution of bone volume in mice.

It is important to note that both inhomogeneous models are not perfectly spherical; instead, they are voxelated all the way to the edges, hence the shape of the marginal voxels is still cubic.

In order to maintain a realistic comparison, for XFI we choose the X-ray beam parameters as if this mouse-like object were scanned with a monochromatic synchrotron beam (like in the aforementioned experimental studies), while for the conventional X-ray imaging, we use the beam parameters from a preclinical small-animal scanner (SmART+, Precision X-Ray, USA) with its intrinsic broad bremsstrahlung spectrum. In both cases, the total absorbed dose per image will be kept the same.

In other words, to directly compare XFI with conventional X-ray imaging, we use exactly the same object and target, as well as the identical total exposure dose. Our comparison may be slightly unfair for XFI, as we apply only one single X-ray detector with its correspondingly small solid angle, similar to the previously mentioned immune cell tracking case. By using only a single detector, the total detection solid angle is on the order of just about 0.1% compared to the full solid angle; however, the sensitivity of XFI remains still sufficient to get statistically significant results. Therefore, by employing more detectors and thus increasing the solid angle, the sensitivity of XFI would be further enhanced (by about the square root of the number of detectors).

For the conventional X-ray imaging, we study three different cases: (1) we measure only a single projection direction, i.e. a pencil beam illuminating only the centre, just as in the XFI setup; (2) a cone beam that illuminates the entire sphere; and finally (3) we apply a full CT scan. In all these three cases, the total absorbed dose is kept the same as in the XFI setup. The following Table 1 gives an overview of the different scenarios.

**Table 1:** Different Scenarios used for Comparison of Significance and Limits for Detectable Marker Mass

Name	Beam Shape	Energy	Imaging Methode	Data Source
XFI	Pencil Beam	53 keV	Single Imaging	Monte Carlo, Analytical Estimate
Scenario 1	Pencil Beam	40 kVp	Subtraction imaging	Monte Carlo, Analytical Estimate
Scenario 2	Cone Beam	40 kVp	Subtraction imaging	Monte Carlo, Analytical Estimate
Scenario 3	Cone Beam Computed Tomography (CBCT)	40 kVp	Subtraction imaging	Monte Carlo, Analytical Estimate
Scenario 4	Cone Beam	40 kVp	Single Imaging	Analytical Estimate
Scenario 5	Cone Beam Computed Tomography (CBCT)	40 kVp	Single Imaging	Analytical Estimate

Note that all XFI scenarios mentioned here are 2-dimensional, but we have shown in the supplementary materials, that a 3-dimensional XFI Scan can be performed using an identical dose and deliver similar significance in the results.

### Transmission and CT

For the transmission simulation, we assumed an X-ray tube positioned 150 mm from the target, as used in the SmART+ system, with a 100 μm focal spot. This setup ensures that the only limiting factor for the resolution is the detector. In the SmART+ system, the detector is located 300 mm behind the centre of the target, resulting in a magnification factor of 3 and a theoretical reconstructed voxel side length of 67 μm. To minimise noise from scattering and because the actual focal spot size in the SmART+ is unknown, the chosen reconstructed voxel side length for CT is set to 100 μm.

In CT, the spatial resolution is approximately the object size divided by the number of pixels in the dimension of interest. Also, detector characteristics will influence the spatial resolution that can be obtained. We assume an optimal detector with no additional noise, no additional blurring and with a sufficient high number of pixels not to deteriorate the natural spatial resolution. In this case only the size of the object and the number of projection angles are relevant. For a 30 mm object, 300 projection angles—typical for SmART+—were chosen, because the number of necessary projection angles scales with the object size divided by the resolution. For all cone beam measurements (CT or transmission), the divergence angle was set to 5.75 degrees, resulting in full illumination of the sphere.

The backward projections were performed using Python's ASTRA toolbox [11]. The spectra were calculated using Spekpy [12]. We assumed a 2 mm aluminium filtration and a 0.8 mm thick beryllium window, with a tungsten anode angled at 12 degrees, and employed an energy of 40 kVp for high soft tissue contrast measurements.

**Fig. 1:**
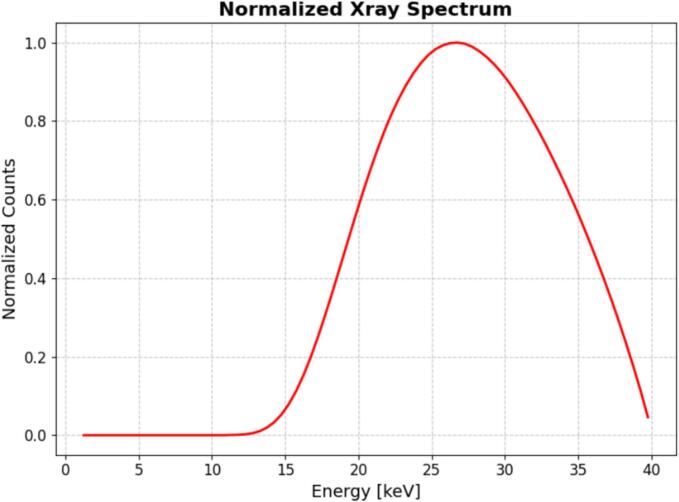
Spectrum of a 40 kVp, tungsten anode under 12° with 2 mm aluminium filter

**Fig. 2:**
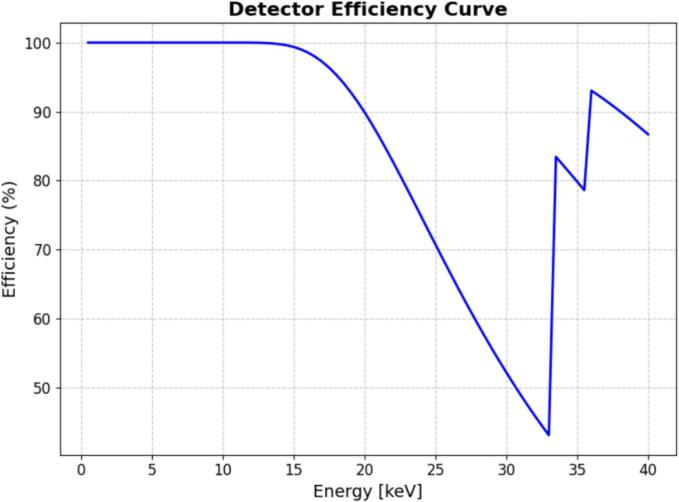
Efficiency curve for a 200 μm thick CsI crystal

For all transmission cases, the following spectrum, depicted in Fig. 1, and efficiency curve, depicted in Fig. 2, were used with a binning of 0.5 keV:

The efficiency for the incident spectrum would be 71.4%, and for the spectrum after passing through 30 mm of water, it would be 66.6%. The calculations for this can be found in the Appendix A, [33]. The low iodine concentration does not significantly alter the spectrum, resulting in no shift in efficiency. For the analytical calculations, the total spectra were each multiplied by the efficiency curve and then summed. Therefore, intensities are given in units of counted photons irrespective of their *energy* as X-ray absorption imaging is mainly done with *photon counting*, while XFI is certainly a *spectroscopic* technique.

In the simplest transmission scenario, we use a pencil beam with an area of 1 mm^2^, thereby illuminating a bit more than the total cross-sectional area of the iodine sphere in the homogeneous scenario and the total iodine voxel in the inhomogeneous scenario.

Note that the efficiency of the total detector decreases at lower energies due to the carbon cover. This cover was not considered in the calculation of the detector’s efficiency curve, as it was included in the transmission calculations and simulations.

However, we must note that this sensitivity level in case of scenario 1, 2 and 3 of the conventional X-ray imaging setup fully relies on the fact that the *object* sphere was taken as perfectly *homogenous* If, as in a real mouse, the object is inhomogeneous, the denominator in the equation for Z is, of course, larger, because, in addition to the quantum noise, there is an extra noise component arising from the inhomogeneity of the object [13,14].

### Whole Body Absorbed Dose Calculations for Photon Number Estimation

In our simulations and analytical estimates, it is crucial to determine the appropriate number of photons for accurate modelling and the comparison with XFI and its absorbed dose like in our mentioned experiments. To ensure a fair comparison, all methods were simulated with the same absorbed dose suitable for in vivo applications [4].

The dose was analytically calculated based on the energy deposition likelihood obtained from Xraylib [15], tracing the path of the primary photons. Notably, this calculation does not account for the dose contributed by scattered photons. However, in scenarios 1, 2 and 3, the additional scattered dose would be consistent, in contrast to the first scenario at 53 keV. In this initial scenario, both the scattering likelihood and the energy of the scattered photons differ slightly. Nevertheless, we consider the resulting effects to be sufficiently minor; therefore, they were excluded from our calculations:

- In XFI, the incoming energy is monoenergetic at 53 keV. Scanning the entire water sphere deposits 0.278 Gy with 1e10 photons per 1 mm^2^ scan point. To calculate this dose and the subsequent scenarios’ doses, the sphere was segmented in the same manner as a raster scan with a pencil beam. For each 1 mm^2^, the length of the path through the sphere was calculated for estimating the absorbed energy along that path. Afterwards, the doses of all voxels were summed to obtain the whole-body dose.

- In scenarios 1, 2, and 3, the lower incoming energy of 40 kVp (which equals a mean energy of 27.39 keV) increases the energy deposition cross-section. Thus, the same dose of 0.278 Gy is reached with 5.0e9 photons per mm^2^ or 3.54e12 photons in a cone beam, translating to 1.18e10 photons per direction in the setup used for the CT. For the sake of simplification, we assume parallel beams in our model, as the angles of incidence remain below 2.88 degrees relative to the beam axis.

All calculations were performed assuming a spherical water target. It is important to note that for all cases except XFI, two imaging procedures are necessary, which effectively doubles the dose for subtraction imaging in scenarios 1, 2, and 3. In scenario 4 and 5, where single imaging is used, the dose is equal to the one for XFI.

## Simulations and Analytical Estimations

### XFI

For a direct analytical estimate on the XFI-sensitivity as compared to the conventional X-ray imaging, we start with the XFI case and the pencil-beam scenario (1).

**Fig. 3:**
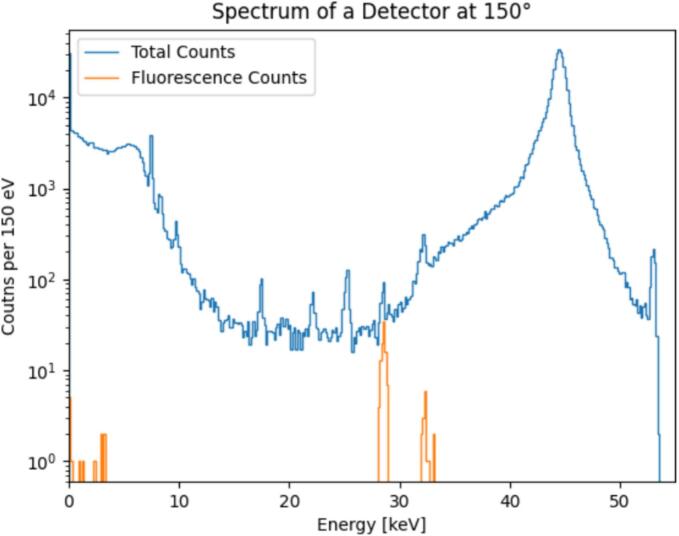
Spectrum simulated for an Amptek SDD (1 mm thick, 50mm^2^ collimated area) under 150° to the incident beam in 6 cm distance to the target with 10^10^ photons per mm^2^ flux of the scanning 1-mm-sized pencil beam. The target is a homogenous water sphere with a 200 ng iodine solution in a 1 mm diameter sphere its centre. Blue is the total recorded spectrum. Orange are those counts created by fluorescence.

In case of XFI the estimate may appear straightforward but it is not trivial, the reason for which is the fact that while calculating the number of detected XFI signal photons is simple, the calculation of the spectral background is complex as it involves calculating the contribution of photons which have been scattered 5 to 6 times by Compton-scattering. Therefore, for taking the spectral background level we use the one from our simulation; the corresponding spectrum is shown in Fig. 3. The total measured spectrum of the detector is depicted in blue. In our simulation, we can easily differentiate between fluorescence and background counts; hence, we can display the actual fluorescence counts in orange. In reality, these would need to be fitted. The position of the detector at an angle of 150° to the incident beam is favourable, as this results in a relatively low spectral background in our signal region [4].

To differentiate signal from noise, there are different methods. On the one hand one can do a scan before the insertion, which would be subtracted from the scan after marker injection. Just like in subtraction CT this is prone to alignment issues and doubles scan time and dose. In X-ray fluorescence there is a second option to do this: K-edge based XFI, which scans with two energies one above and one below the K-edge of the marker. But the doubling of dose and time still remains. On the other hand, one can use the continuity of the Compton background to fit it with a polynomial based on the points outside the signal region, therefore eliminating the necessity of a second scan altogether. This is possible if the number of fluorescence counts is high enough and the fluorescence energy is sufficiently far from the Compton peak in the spectrum.

Apart from the spectral background issue, the fluorescence yield is straightforward, one simply calculates the transmission of the incident synchrotron beam (1e10 photons of 53 keV with a beam size ($A_{B}$) of 1 mm^2^) until the centre of the object sphere and then calculates the number of emitted fluorescence photons, here only for K_α1_ and K_α2_. K_α1_ are the transitions of an electron from the L1 level to the K-shell, while K_α2_ comes from the transition from the L2 level. Since both lines are close to each other, one can take their mean attenuation of the way to the detector.

By also factoring in the aforementioned solid angle (Ω) and the detection efficiency (ε) (1mm-SDD, approximately 27%, based on the photo absorption cross-section of silicon at the energy between the K_α1_ and K_α2_ lines), one arrives at the number of expected fluorescence photons detected.

The simulation can be verified by using an analytical estimation based on values from Xraylib. The combined fluorescence cross-section (σ) for the iodine K_α_ lines is 6.07 cm^2^/g.

For the transmission calculation, the NIST database [16] was used to determine the linear attenuation coefficients of compounds. The incoming transmission ($T_{\mathrm{in}}$) is calculated to be 72% using 15 mm of water with a density of 1 g/cm^3^ and a linear attenuation coefficient $(\frac{\mu }{\rho }$) of 0.22 g/cm^2^ at 53 keV. The outgoing transmission ($T_{\mathrm{out}}$) is calculated in the same manner, but for the lower energy of the K lines, employing an attenuation coefficient of 0.419 g/cm^2^ at 28.5 keV, which results in a transmission of 53%. This leads to an expected value of 143 ± 11 photons.

$$N_{\mathrm{fluorescence}}=\frac{N_{0}\cdot T_{\mathrm{in}}\cdot T_{\mathrm{out}}\cdot \sigma \cdot M\cdot \Omega \cdot \varepsilon }{A_{B}}$$

We can see that our signal scales linearly with the number of incident photons and therefore with dose, if the photon energy remains constant. However, augmenting the number of registered photons by increasing either the quantity or dimensions of the detectors, thus expanding our solid angle, could correspondingly enhance our signal.

A useful measure for the detection sensitivity is the statistical significance Z, which for large enough photon numbers is simply Z = $\frac{S}{\sqrt{B}}$. In this equation, S represents the signal photon counts, while B denotes the spectral background level within the signal range defined by the detector energy resolution. The statistical noise is hence given by the square root of B and Z is expressed in units of one standard deviation (σ). Since both a larger detector area and a higher number of incident photons increase signal and noise alike, the significance scales with the square root of the detected photon count. If we do not limit ourselves to a single detector in the XFI case, the significance could be increased by a factor proportional to the square root of the number of detectors. The theoretical limit for this increase would be a full 4π detector.

Now we can compare our estimate to the results of our Monte Carlo simulation. Here the number of fluorescence photons is 151 ± 12, the error represents the statistical Poisson error. It Is important to note that some fluorescence is also generated by scattered photons; however, these are not included in this estimation and may account for the remaining difference between the simple analytical estimation and the Monte Carlo simulation.

Using 200 ng of iodine, the significance Z of the results from the Monte Carlo simulation in the homogenous case is calculated as follows:

$$Z=\frac{151}{\sqrt{273}}\approx 9.14$$

In the inhomogeneous XFI case the signal photon counts are reduced from 151 to 91, i.e. by 60%. The background is decreased from 273 to 264 photons (96%) because the higher density and higher effective atomic number reduce the transmission while also increasing the Compton scattering.

Therefore, we have a lower significance of 5.60. This is not due to the inhomogeneity, but due to the increased density of bone tissue (1.85 g/cm^3^) compared to the one of water (1.0 g/cm^3^) used in the homogeneous model leading to higher attenuation of the signal photons. In total, this explains the reduction in Z.

To verify our thesis, that the inhomogeneity is not the source of our significance loss, we create another scenario. We look at a perfectly homogeneous sphere made out of 90% soft tissue and 10% bone with the 200 ng marker mass at the centre. The expected number of detected X-ray fluorescence photons in this scenario is 125 ± 11 (based on an analytical estimate) or 120 ± 11 based on the Monte Carlo simulation. This would result in a significance Z of 7.83 from the Monte Carlo simulation.

Comparing both homogeneous cases, we find that the decrease in significance results from higher outgoing attenuation rather than an increase in background photons in the signal region. The signal decreases from 151 to 120, primarily due to the increased attenuation of the outgoing path. The number of background photons in the signal region actually decreases from 273 to 235.

As shown in the supplementary materials, the transmission remains similar for the incoming energy in water compared to a 10% bone/90% tissue mixture. Therefore, the difference in signal arises from the attenuation of the outgoing path.

We recall that in the inhomogeneous sphere the number of X-ray fluorescence photons was 91. This decrease was due to a higher outgoing attenuation, as the incoming attenuation was lower in the inhomogeneous case (than in our new homogeneous case) and thus cannot account for the decrease. This loss in significance could be reduced by using more than one detector, which would result in higher overall significance and higher independence of a single outgoing attenuation path.

The difference in significance between the homogeneous case and the inhomogeneous case, both with 10% soft tissue and 90% bone, is also easily explained due to the voxelization. The outgoing path would need to be attenuated by a composition that is equivalent to 15% bone and 85% soft tissue. Due to our voxelization, this corresponds to a path through 2.25 bone voxels instead of the expected 1.5, resulting in a difference of less than 1 voxel above average, which is indeed possible with our random distribution of bone voxels.

The underlying attenuation calculations can be found in the supplementary materials. There, we have also demonstrated, that a 3D XFI scan utilizing an algorithm similar to the tomosynthesis “shift and add” method makes X-ray Fluorescence Tomosynthesis (XFTS) possible with an identical dose while maintaining similar significance (Z = 8.8 instead of 9.1 in the homogeneous case). This is in contrast to XFCT, where significant measurements are required for every projection angle, thereby increasing the dose proportionally to the number of projection angles.

### Scenario 1: Pencil Beam Subtraction Imaging

For the pencil beam scenario in the conventional X-ray imaging the estimate is much simpler: we need to calculate two sub-scenarios, one with the target sphere and the other without, that is, with and without the iodine. Therefore, the contrast, as defined above, is simply given by the corresponding intensity difference $I_{1}-I_{2}$ compared to the statistical photon number noise. This comparison shows the fundamental difference between both methods:

In conventional X-ray imaging, the sensitivity is limited by the ability of measuring the intensity difference in transmission direction with sufficient statistical significance. The challenge here is that most incident photons are transmitted, leading to a relatively large denominator in the equation for Z, while the numerator, the intensity difference, is rather small.

In contrast, in XFI the background noise (i.e. the denominator) is relatively small as the background is little if the incident photon energy is chosen far away from the K-edge This makes it less likely for an incident photon to undergo a sufficient number of Compton scattering events to reach the XFI signal range. Furthermore, aside from the spectral background from multiple Compton scatterings, there is only the fluorescence line on top; thus, one does not need to compare this signal with a second signal as is the case with the two intensities in conventional X-ray imaging.

In this pencil beam scenario, the relevant area on the detector is 1.2 mm by 1.2 mm due to the size of the pixels (200 µm). Therefore, some scattered photons will always be counted as signal counts. While this effect is negligible in the pencil beam scenario (1), it slightly reduces the significance in the transmission scenario (2) and (3).

For the analytical estimate of the transmission cases, we can simply calculate the transmission with and without the 200 ng marker. In the inhomogeneous case, this is straightforward due to the voxelization of the model. For this, the transmission for each energy bin through a known amount of bone and soft tissue is calculated, whereby *μ*/*ρ* is the density-dependent mass attenuation coefficient. The diameter of the sphere is 3 cm. The distance between the source and detector, after subtracting the target's depth, is 42 cm. The thickness of the carbon fibre plate is 0.075 cm and ∊isthe efficiency of the detector for each energy, 90% is the percentage of soft tissue in the target and 10% the amount of bone.

$$N=N_{0}\sum_{energies}e^{-3.0\left[cm\right]\cdot \frac{\mu }{\rho }\left(\text{softtissue}\right)\cdot 1\left[g/cm^{3}\right]\cdot 90\%}\cdot e^{-3.0\left[cm\right]\cdot \frac{\mu }{\rho }\left(\text{bone}\right)\cdot 1.85\left[g/cm^{3}\right]\cdot 10\%}\cdot e^{-0.075\left[cm\right]\cdot \frac{\mu }{\rho }\left(\text{carbon}\right)\cdot 2.2\left[g/cm^{3}\right]}\cdot e^{-42\left[cm\right]\cdot \frac{\mu }{\rho }\left(\text{air}\right)\cdot 0.00129\left[g/cm^{3}\right]}\cdot \;∊$$

$$N_{\mathrm{Iodine}}=N\cdot e^{-0.1\left[cm\right]*200e-6\left[g/cm^{3}\right]\cdot \frac{\mu }{\rho }\left(\text{iodine}\right)\cdot \;\rho }$$

$$Z_{\mathrm{Inhomogen}}=\frac{\left|N_{\mathrm{Iodine}}-N\right|}{\sqrt{N_{\mathrm{Iodine}}+N}}=5.72$$

This is less than our value from the simulation of Z = 7.85, as the amount of bone in the pencil beam simulation path is less than the average amount. Therefore, we will now analytically calculate the effect of different bone to soft tissue ratios on the significance. If we use an estimate that considers only soft tissue in that path, we get a better analytical estimation:

$$Z_{\mathrm{Inhomogen}}=7.67$$

**Fig. 4:**
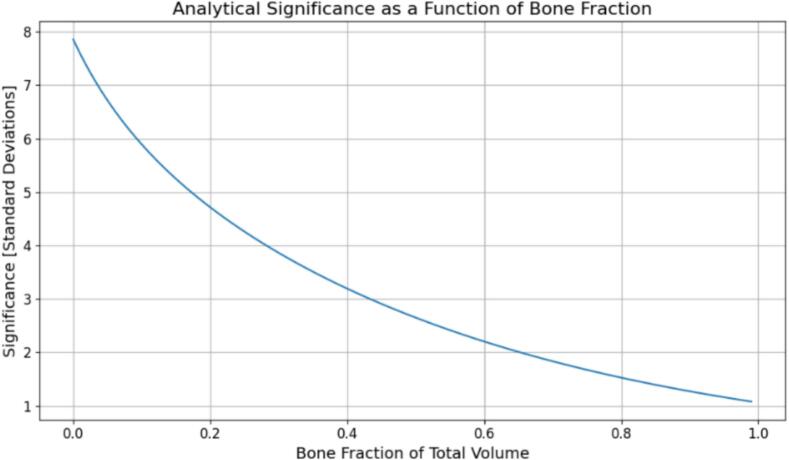
Analytical calculation of the significance of the subtraction transmission images based on the fraction of bone in tissue.

In general, however the position of the marker is not known and therefore an orientation that favours the least amount of bone in the path through the marker is unlikely to be chosen at random, therefore the approximation with 10% bone in the path is better suited to get a general idea for random inhomogeneous objects. This results in a Z of 5.72 on average and 4.58 for 20% percent bone as can be seen in Fig. 4:

We can also observe that the measurement can no longer be considered significant (Z > 3) [17] when the fraction of bone in the beam surpasses 40%. In this inhomogeneous model, such a condition is statistically rare; however, in the case of a mouse, certain paths through the skull or pelvic bones could exceed this threshold.

In the homogeneous case, the analytical estimation is more complex because both the target and the marker volume are spherical: We must calculate each possible path individually due to the curvature of the marker solution and the water sphere. The average length of the path through the water sphere using the pencil beam is approximately 29.99 mm, while the average path length through the marker sphere is 0.53 mm. However, the density of iodine is increased by a factor of $\frac{6}{\pi }$ which corresponds to a value slightly greater than double the original density due to the same mass (200 ng) being contained in a smaller volume — a sphere rather than a cube.

The calculation is otherwise equivalent, and the resulting significance is Z = 7.65, calculated using 10 000 possible paths through the sphere with a 1mm x 1mm pencil beam directed in the z direction and x, y coordinates in the range [-0.5, 0.5] mm. The choice of 10,000 paths was made to ensure sufficient confidence in accurately determining the sphere’s diameter, while the [-0.5, 0.5] mm range was selected to emulate a 1 mm^2^ pencil beam as used for synchrotron-based XFI-measurements.

In the homogenous case the analytical estimate of 7.65 is very close to our simulation result of Z = 7.60, demonstrating consistency between the analytical and computational approaches.

### Scenario 2: Cone Beam Subtraction Imaging

**Fig. 5:**
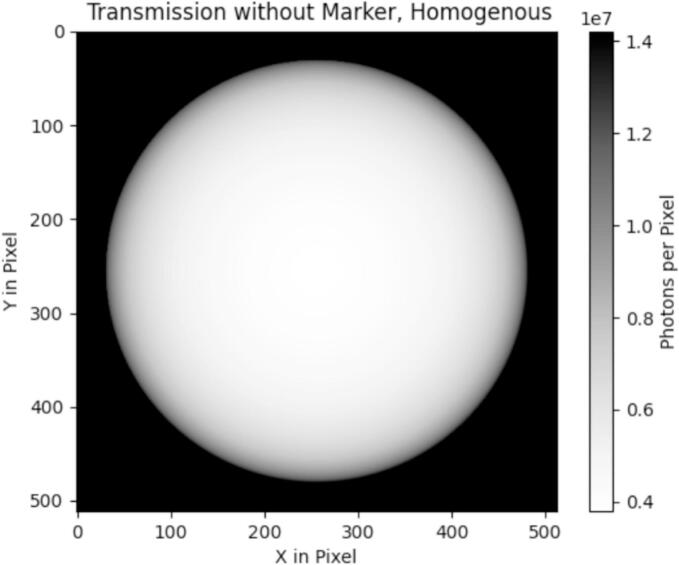
Transmission image of the simulation of the homogenous sphere without marker

**Fig. 6:**
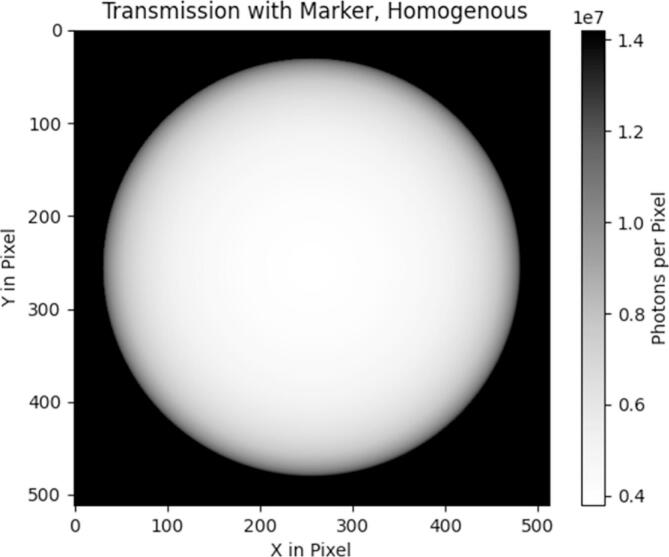
Transmission image of the simulation of the homogenous sphere with iodine marker in the centre. There is no visible difference to the picture without marker (Fig. 5).

In this scenario, we conducted two Monte Carlo simulations for the homogeneous case (Fig. 5, Fig. 6) and two for the inhomogeneous case (Fig. 9, Fig. 10), for each case one with and without 200 ng of iodine for subtraction imaging with a cone beam. The main difference to scenario 1 is that the whole sphere is illuminated at the same time, so scattering becomes more important. In addition, in the cone beam case the beam has a slight divergence which becomes apparent in the edges of the images of the inhomogeneous voxels.

**Fig. 7:**
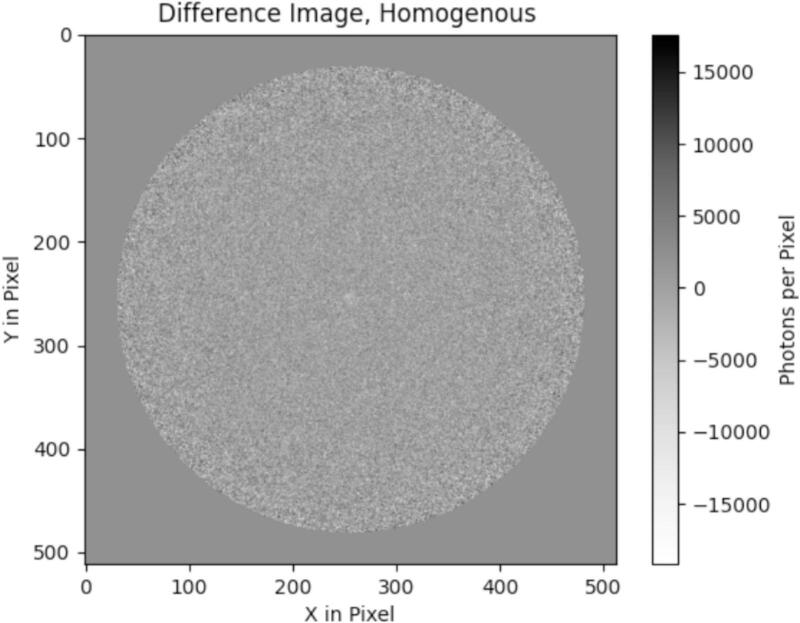
Difference image of the images with and without marker. In the centre, the iodine solution becomes visible.

**Fig. 8:**
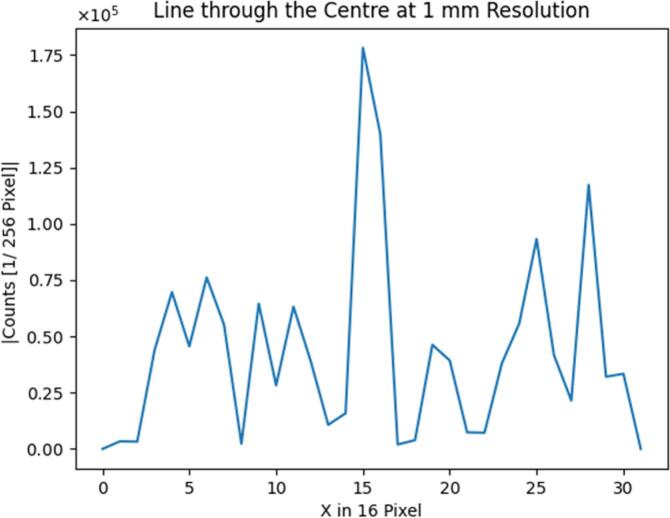
Absolute value of the sum over 1 mm pixels through the centre line of the difference image of the homogeneous model.

In the homogeneous case, the entire sphere was illuminated by a cone beam, and the photon count over the relevant area of the pixel detector is depicted in Fig. 5, Fig. 6. While these figures are nearly identical, Fig. 6 contains an additional, albeit on its own invisible, amount of tracer—the iodine solution—located at the centre of the water sphere. This inconspicuous presence of the tracer, despite being undetectable in the figure, is significant for understanding the transmission characteristics being analysed. By subtracting the image without the tracer, we obtain Fig. 7, where the marker solution becomes visible. This can be quantified by summing over 16 by 16 pixels of the subtraction image along the centre line, as depicted in Fig. 8 which is equivalent to 1 mm^2^ of the target model. The calculation of the significance Z = 8.45 follows later on.

**Fig. 9:**
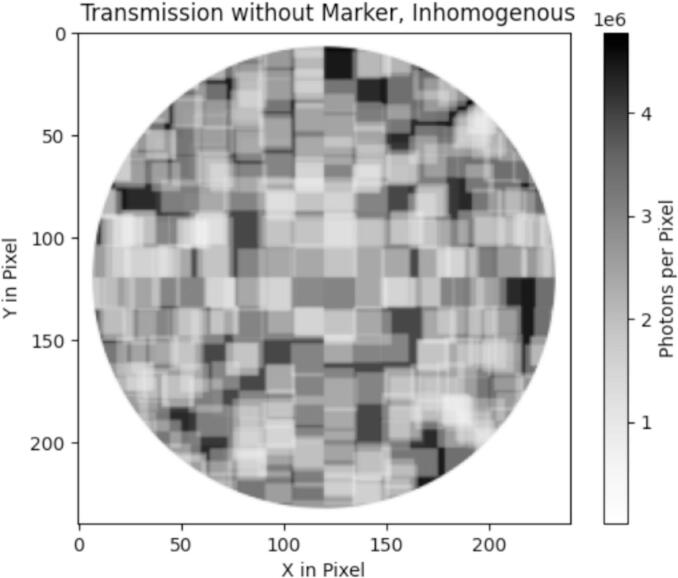
Transmission image of the simulation of the inhomogeneous sphere without marker

In Fig. 9, we present the equivalent simulation for the inhomogeneous model using binary voxels, representing soft tissue at 1 g/cm^3^ and bone at 1.85 g/cm^3^. In this case, we utilised half the divergence of the photon beam because the high photon count and the large data structure in the simulations for the homogeneous case proved to be very computationally intensive. This approach is valid because the scattering of photons from the edge of the sphere can be neglected when examining the image of the centre—especially in comparison to the transmitted photons. Therefore, only the central quarter of the area was illuminated. This approach results in a perfectly round shape, as the beam itself is circular. To confirm that inward scattering is indeed negligible, we compared it to outward scattering, which is expected to be similar in magnitude. As shown in Fig. 11**,** the differences in outward scattered photons at the edges are nearly zero and exhibit more homogeneity than the variations within 1 mm^2^ closer to the centre.

**Fig. 10:**
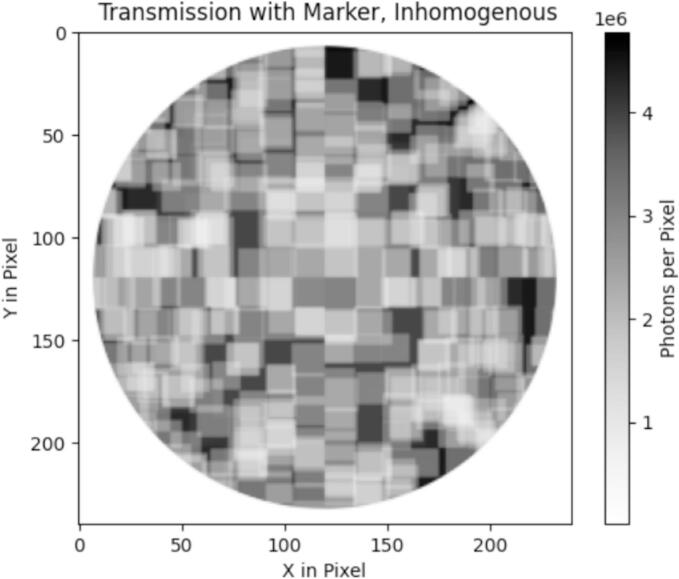
Transmission image of the simulation of the inhomogeneous sphere with marker. There is no visible difference to the picture without marker (Fig. 9).

**Fig. 11:**
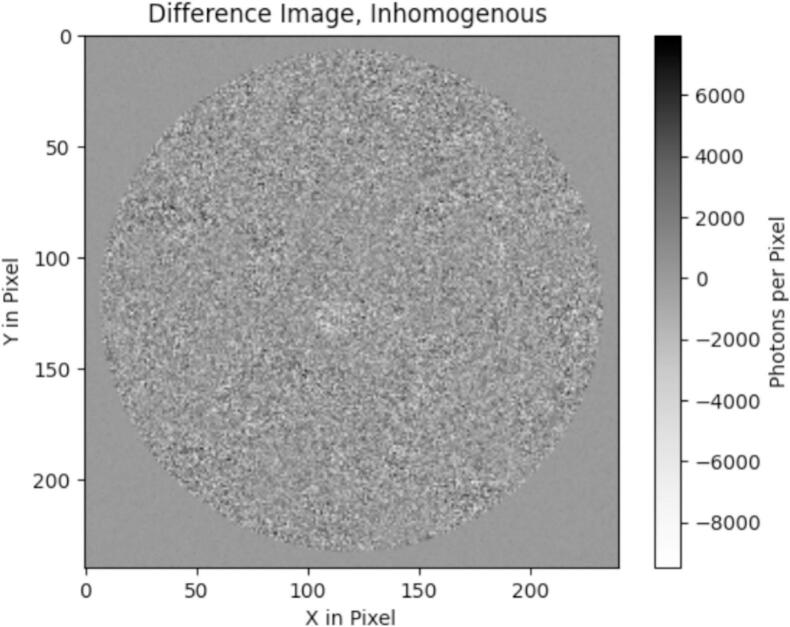
Difference image of the images with and without marker. By taking the difference of the two pictures, the iodine solution becomes slightly visible in the centre.

In Fig. 10, we depict the same model and simulation parameters, but this time with the iodine marker placed in the voxel at the bottom left of the centre. Due to the voxelization of a sphere with a diameter of an even number of voxels, the z-axis is does not run through a central voxel row, but all neighboured central voxel rows are placed around the z-axis. Although the marker is present, it is again not visible on its own, particularly against the surrounding tissue that, due to statistical variations, contains slightly more bone. The distortion of pixels closer to the edge results from the divergence of the beam and would not be observed in a pencil beam scan.

**Fig. 12:**
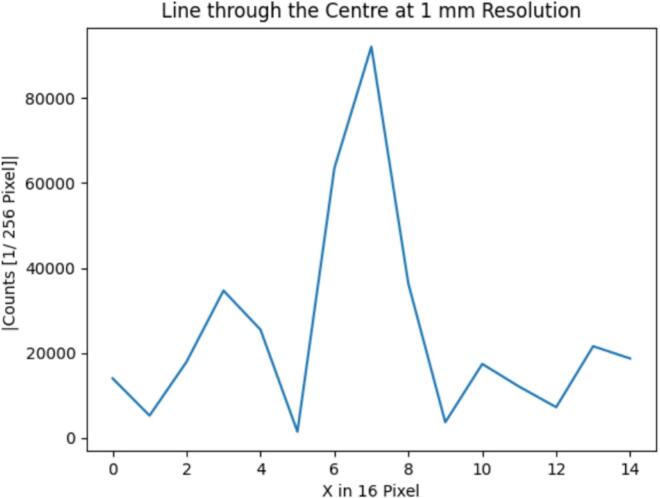
Absolute value of the sum over 1 mm pixels through the centre line of the difference image of the inhomogeneous model.

Fig. 11 shows again the subtraction image but now with visible marker position. This can be quantified again by summing over 16 by 16 pixels of the subtraction image along the centre line, as depicted in Fig. 12. In both scenarios, a significant peak (Z= 7.23) is observed at the centre where the marker is positioned. This detection was made possible by the increased statistics resulting from summing over an area of 1 mm^2^ of the sample (equivalent to 3.2 x 3.2 mm on the detector). In contrast, no peaks were visible in the 100 µm resolution paths.

Subsequently, we calculated the significance at the position of the marker for both cases. The calculated significance values were found to be 7.23 for the inhomogeneous case and 8.45 for the homogeneous case:

$$Z_{homogeneous}=8.45\;$$

$$Z_{inhomogeneous}=7.23$$

For this analysis, the sum of the central 16 by 16 pixels was selected, as they correspond to the central 1 mm by 1 mm of the sample, taking into account the magnification of the cone beam. In the inhomogeneous case, the marker appears slightly off-centre due to the voxelization of a 30 mm object with 1 mm voxels. Therefore, the transmission is slightly higher in the inhomogeneous case.

It is crucial to note that achieving these significance values requires perfect alignment of the targets. This was made possible due to the identical density and material distribution of both targets used in the simulations, which is most likely unattainable in reality, especially in vivo. Additionally, it would still be challenging ex vivo due to changes in the tissue over time. But most important the dose in subtraction imaging is double the dose of single imaging or XFI.

If we take motion into account, we get a scenario with basically two different inhomogeneous spheres. We can see in the supplementary materials that, with these, the results are no longer significant

### Scenario 3: CT Subtraction Imaging

In the CT reconstruction, 300 images were used, which corresponds to a spatial resolution of 100 μm and is a typical amount for such measurements with a SmART+. However, the resolution was decreased in the CT to 1 mm^3^ voxels to denoise the images and facilitate better comparisons with the other methods that have similar resolution.

**Fig. 13:**
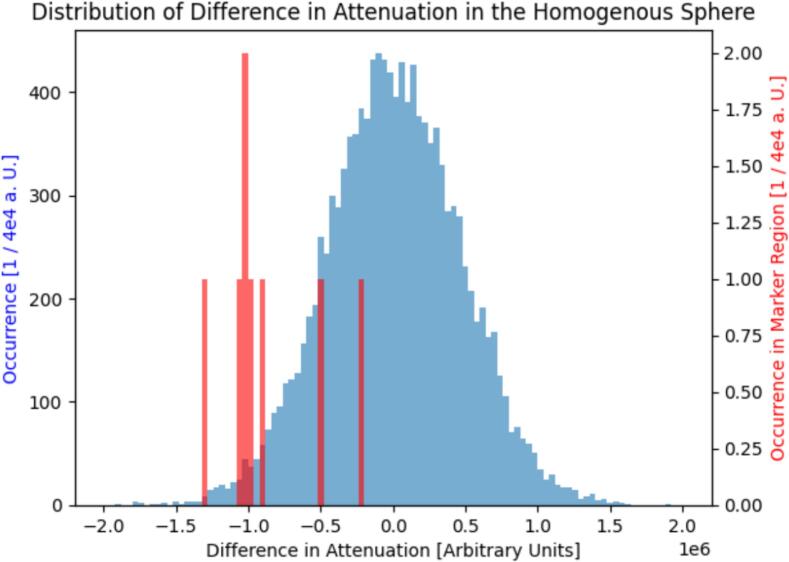
Histogram of the difference in attenuation between the CTs with and without the marker, highlighting in red the region around the centre where the marker was placed.

**Fig. 14:**
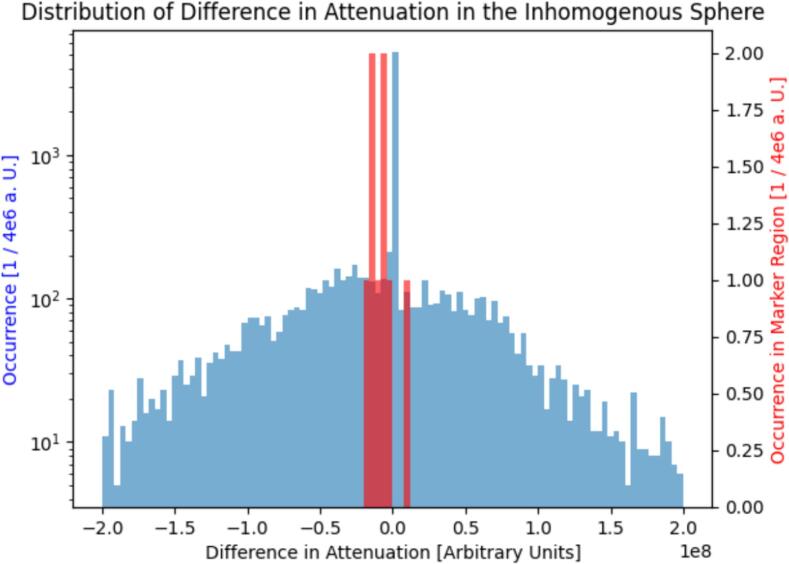
Histogram of the difference in attenuation between the CTs of the inhomogeneous sphere with and without the marker, highlighting in red the region around the centre where the marker was placed.

Fig. 13 for the homogeneous case and Fig. 14 for the inhomogeneous case.

The significance results from the CT are too low to determine the marker position with confidence. In the homogenous case, the 8 central voxels had, on average, a difference in attenuation that was 1.9 σ away from the mean attenuation difference.

In the inhomogeneous CT case Fig. 14, the fluctuations in attenuation difference due to noise were greater than those created by the marker. In the general fluctuations we have a peak at the centre of the histogram. This central peak arises from the difference between two identically reconstructed voxels and is indeed very close to 0, as expected compared to the noise. The tails on either side are caused by differences created by shot noise, so differences due to the statistical nature of the scattering and not due to differences in transversed tissue. In this case this was amplified in the reconstruction due to the inhomogeneity and overall lower transmission. Note that the overall occurrence (how many voxels have a certain difference in attenuation) is on a logarithmic scale here.

The individual significance of the subtraction image for each angular transmission angle is significantly lower than that from a single direction using 300 times the photons. To achieve a depth resolution of 1 mm, only 30 transmission angles are necessary so we can combine 10 directions in order to improve statistics. Consequently, each combined angle receives a photon count that is a factor of 30 lower than that of the single-direction measurement. This reduction in photon count leads to a decrease in significance, which can be quantified by the square root of the photon factor, $\sqrt{30}$.

Given our significance value of 8.45 for the transmission images of the homogeneous sphere, we anticipate a reduction in significance to approximately 1.54 (calculated as 8.45 / $\sqrt{30}$). This value aligns with the measured significance of 1.9 obtained from the CT simulation. In the inhomogeneous case, this estimate was not conducted, as each angular image exhibits a different significance. Each direction has a wide variety in transmission due to the differing amounts of bone in various locations, ultimately reducing the significance of most angular images far below a meaningful threshold.

### Comparing the Subtraction Imaging Scenarios with XFI

In order to evaluate the efficacy of different imaging techniques, we conducted a comparative analysis of the performance of XFI and subtraction X-ray imaging methods in both homogeneous and inhomogeneous spheres. Table 2 presents the results of our simulations, specifically comparing the performance in the two sphere types. The data indicate that all methods, except for conventional CT, can achieve satisfactory results in the homogeneous sphere. However, in the inhomogeneous case, there is a slight reduction in significance across the methods. This decrease can be attributed to the reduced transmission resulting from the introduction of bone into the imaging scenario. that is, the object before and after adding the contrast agents are identical except for the single one voxel containing the contrast agent. However, below we will also treat the case where one does not make a before/after-comparison, but just a single measurement. The above results are just for a first comparison to XFI, which, as mentioned above, is in the signal size mostly independent of the inhomogeneity of the scanned object, only the noise is directly dependent on the homogeneity of the object which creates an overall dependency of the significance.

**Table 2:** Significance values calculated using perfect positioning and (except for XFI^1^) subtraction imaging with two identical objects modulo the addition of the marker.

Z for 200 ng Iodine	Inhomogeneous Sphere	Homogeneous Water Sphere
XFI^a^	5.60	9.14
(1) 40 kVp pencil beam^b^	4.58 - 7.85	7.60
(2) cone beam transmission^c^	4.58 - 7.23	8.45
(3) full CT	0.16	1.90

a All XFI significances were obtained using only one single detector covering about 0.1% of the full solid angle and could be easily improved by employing additional detectors.

b This value depends strongly on the orientation of the inhomogeneous object and the resulting amount of bone in the path containing the marker. A lower value is expected for a typical high amount of bone (20%), while a higher value is anticipated for a typical low amount of bone (0%).

c The inhomogeneous case here is analogous to the situation observed with the pencil beam.

But to fairly compare both imaging techniques (XFI and classical X-ray imaging) we have to consider the radiation dose, the dose for conventional imaging methods is doubled when using two images (which is also true in K-edge imaging). When adjusting for this by applying a correction factor of $1/\sqrt{2}$ to the significance values of the transmission cases, this further alleviates the higher significance in XFI and it emerges as the better option for in vivo imaging of iodine marker masses as low as 200 ng within a single voxel. In addition, the XFI-based sensitivity in terms of Z can be easily increased by using more than just one single detector as mentioned above.

To address the limitations of transmission imaging, a potential solution is to utilize a single image instead of relying on subtraction techniques. This approach will be explored in the next chapter, particularly in relation to the challenges posed by the inhomogeneous nature of biological tissues, where variations in tissue attenuation contribute significantly to an increase in the required marker concentration.

### Required Amount of Marker for Single Image in Transmission Imaging and CT

In single conventional imaging, a mass of 200 ng is impossible to find so instead of calculating its significance we instead calculate a mass that would be visible.

When analysing a sample, determining the position of a potential marker can be approached in two ways, especially when utilising a single transmission image to reduce dose and motion artefacts compared to subtraction imaging. However, this approach necessitates a higher concentration of the marker to significantly affect the transmission, surpassing the inherent attenuation caused by tissue inhomogeneities.

**Fig. 15:**
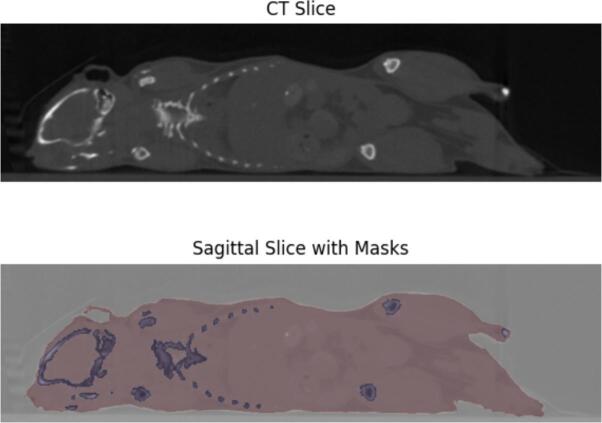
CT of a mouse (top) and CT with overlay (bottom) of the contouring of the whole body (red) and the bones (blue).

The model for attenuation, which previously considered only soft tissue with a density of 1 g/cm^3^ or cortical bone at 1.85 g/cm^3^, is no longer sufficient. While we could attempt to integrate additional tissue types and densities to improve the accuracy of the expected attenuation coefficients in a mouse model, this method would consistently yield inferior results compared to using the attenuation distribution derived from actual mouse data. By discarding the need to differentiate between various tissue types, we can omit µ/ρ and ρ for a voxel. Instead, we can focus directly on the μ value of a voxel for individual tissues, which can be acquired from the Hounsfield Units (HU) of a mouse CT scan (Fig. 15). The mouse imaged was a SCID-mouse (CB17/Icr-Prkdcscid/IcrIcoCrl Charles River, Wilmington, MA, USA), examined within an approved animal testing protocol (permission number N029/2023, approved by the local authorities of the city of Hamburg). This image was acquired with a SmART+ scanner installed at the University Hospital Hamburg-Eppendorf (Hamburg, Germany), using a protocol for high soft tissue contrast and low noise (flat panel fluoroscopy with 360° rotation, 40 kVp, 0.8 mA, acquisition time of 60 seconds, and a reconstruction grid of 100 µm). The conversion from transmission images into HU was performed according to the manufacturer’s calibration protocol. The contouring of the body and organs was performed with a dedicated small animal treatment planning SmART ATP system from SmART Scientific Solutions B.V. (Maastricht, The Netherlands).

**Fig. 16:**
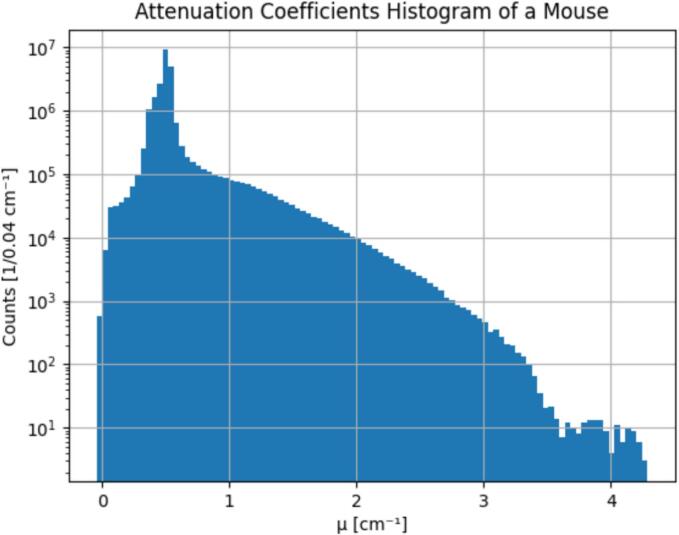
Histogram showing the distribution of attenuation coefficients for our mice model at 0.1 mm resolution, based on conversion from a CT image of a mouse.

**Fig. 17:**
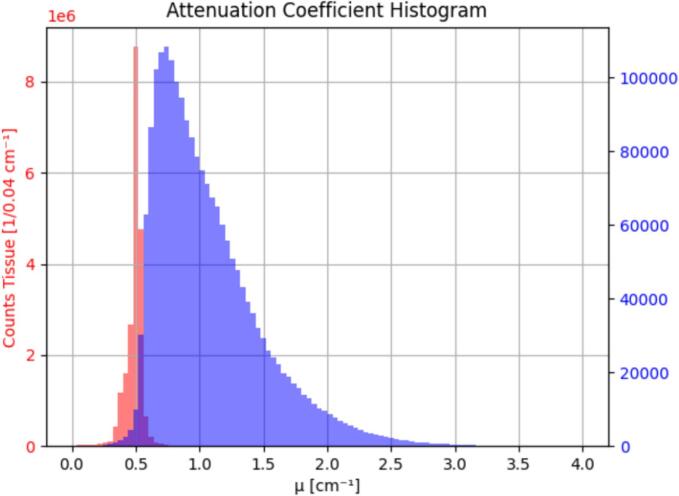
Histogram depicting the differences in attenuation coefficients between soft tissue and bone in a mouse at 0.1 mm resolution, based on conversion from a CT image of a mouse.

To calculate the required concentration for this marker, we first need to obtain the linear attenuation coefficients derived from CT scans. This distribution was calculated using the maximum resolution of 100 µm voxel length. We converted the Hounsfield Units (HU) back based on the calculated X-ray spectrum generated by the SmART+ system operating at 40 kVp. Utilizing the conversion formula with $\mu_{\mathrm{water}}$ calculated with the energy weights of the spectrum,

$$\mu =\mu_{\mathrm{water}}\times \left(\frac{\mathrm{HU}}{1000}+1\right)$$

we can derive the μ distribution in Fig. 16:

If we want to separate tissue from bone, we must use the contoured CT, as the regions overlap. The results are shown in Fig. 17. Notably, the attenuation properties of soft tissue closely align with a standard density of 1 g/cm^3^. In contrast, the distribution for bone exhibits significantly broader variability; its tail approaches 3.5 1/cm, which corresponds to a density of 1.85 g/cm^3^.

This broader distribution can be attributed to several factors:

1. Most mouse bones are hollow, with lower-density marrow.

2. The small size of the bones often results in voxels being incompletely filled with bone.

3. Some bone structures are located near the surface, including elements such as teeth and air, which can visually be categorized as bone while exhibiting lower attenuation values.

### Scenario 4: Cone Beam Single Imaging

**Fig. 18:**
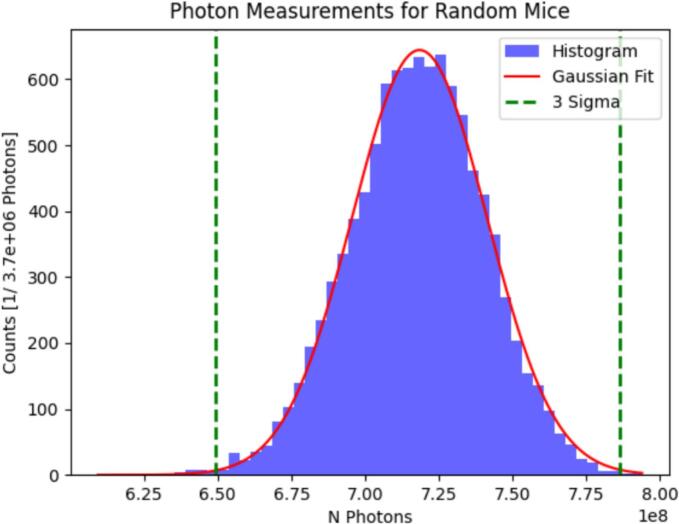
Histogram demonstrating 10 000 different transmission samples through randomly chosen voxels of a mouse, featuring a Gaussian fit and marked vertical lines at the 3-σ interval.

With the attenuation coefficients established, we can proceed to analyse the transmission case for a randomly generated transmission path. To achieve this, we created 10 000 samples of 300 voxels, each with a depth of 100 μm, and calculated the transmission through these tissues (see Fig. 18). These data were fitted with a Gaussian curve, and the 3σ environment is marked.

The mean number of photons calculated to be measured by the detector is 7.18e8, based on 5e9 initial photons, while accounting for Poisson noise, the product of transmission through the 300 voxels, and the detector efficiency. The distribution can be very well fitted with a Gaussian distribution, resulting in a very good fitting coefficient of determination (R^2^) value of 0.997 and a standard deviation of 2.28e7.

To be reasonably certain that a measurement involving the marker is indicative of its presence rather than due to inhomogeneity, we need a sufficient mass of iodine (M) to reduce the photon count by 3 σ from 7.18e8 to 6.49e8, which amounts to approximately 56 µg of iodine. This threshold ensures that the marker's influence is significant enough to differentiate it from background noise and variability in the tissue structure. It is calculated using the Lambert-Beer law, incorporating the linear mass attenuation coefficient per density of iodine ${\left(\frac{\mu }{\rho }\right)}_{\mathrm{iodine}}$ multiplied by the mass per voxel $\frac{M}{1\left[mm^{3}\right]}$ and the voxel length:

$$N-3\sigma =6.49\times 10^{8}=7.18\times 10^{8}\;\cdot exp\left(-{\left(\frac{\mu }{\rho }\right)}_{\mathrm{iodine}}\cdot \frac{M}{1\left[mm^{3}\right]}\cdot 0.1\left[cm\right]\right)$$

In other words, 56 µg of iodine contained within a 1 mm^3^ voxel is sufficient to provide reasonable certainty that the transmission will be reduced by an amount unlikely to be attributable to tissue inhomogeneity, particularly due to variations in the amount of bone present along the beam path.

There are two reasons why this was only verified analytically. The first is that to achieve the same statistics, we would need to perform 1e4 Monte Carlo simulations with 5e9 photons, which would require a significant amount of computing time and resources (39% more than all other simulations in this study combined). The second, more pertinent reason is that we have demonstrated for very low amounts of marker that our analytical estimates match the simulations. Therefore, we can extrapolate that with higher amounts of marker, the estimates should be even more accurate. This estimate holds true under the assumption that the introduction of iodine does not replace the existing tissue but is instead absorbed, resulting in an increase in overall density without altering the volume. For sufficiently low masses, this is a reasonable assumption, as the mass of the iodine constitutes only 5.6% of a tissue volume of 1 mm^3^.

If we assume full replacement of tissue by iodine, then the iodine must account not only for its own contribution to attenuation but also for the loss of transmission caused by the absence of the replaced tissue. Given an expected photon count of 7.23e8 for 29.94 mm of tissue, reducing this to 6.49e8 requires an iodine mass (M) of approximately 59 µg.

The determination of which estimate serves as the better approximation depends on the form in which the iodine is present, a discussion that is outside the scope of this analysis. However, we can be reasonably confident that the optimal approximation for the required iodine mass lies in the range of 56 to 59 µg which is significantly higher than the previously estimated 200 ng. This increase is anticipated, as the earlier estimate solely considered statistical noise between two measurements along the same transmission path, leading to diminished confidence in accurately identifying the marker. In this scenario, the inherent inhomogeneity of the target contributes to variability between measurements that is orders of magnitude greater than the statistical noise considered previously. As a result, the number of photons needed to confirm the presence of the marker may actually be lowered because, at such high concentrations, the required marker levels become relatively insensitive to fluctuations caused by shot noise. Therefore, while a greater concentration is necessary for detection, its substantial presence helps to mask the impact of random noise, allowing for a more reliable identification of the marker.

### Scenario 5: Single Imaging CT

An alternative approach would be to utilise CT imaging instead of a transmission image in order to decrease the variance in measurements. In this scenario, it is sufficient for only one voxel to contain enough marker to compensate for the difference in attenuation between the average soft tissue and the highest attenuation found in bone. For this analysis, we rescaled the CT image of a mouse to a 1 mm voxel size to match the spatial resolution previously employed in our assessments. Notably, the highest attenuation of a 100 µm voxel is above 3.5 1/cm; however, the 1 mm voxel corresponding to our sample data—specifically a molar tooth—yields a mean value for µ of 2.65 1/cm. In contrast, the average µ for soft tissue is only 0.49 1/cm, resulting in a difference in attenuation of 2.16 1/cm which is equivalent to that of 123 µg of iodine.

This increase compared to the 56 µg can be attributed to the reduction in voxel size, which does not affect the average attenuation for soft tissue. However, with a more uniformly sampled dataset, bones are more likely to be adjacent to other bone voxels. This proximity enhances the edge cases in µ or transmission within this lower-resolution three-dimensional sample, thereby amplifying the overall variance in attenuation measurements. Sampling the transmission scenario earlier from the 1 mm mouse yields an almost threefold increase in the standard deviation, resulting in a threshold exceeding 150 µg in order to surpass the 3σ environment for transmission as well.

### Dose comparison between XFI and PET/SPECT

As mentioned above, PET/SPECT offer the highest detection sensitivity, but XFI offers some advantages in terms of accessible information to be retrieved from an XFI-scan. However, here, we now want to compare the absorbed dose levels, including the ones from PET/SPECT.

Molecular imaging today is mainly feasible using nuclear medical imaging approaches like SPECT or PET. The assessment of exposure to ionising radiation for the small animals has also been done in comparison to those exposures in PET or SPECT imaging. In this case, calculations on simple phantoms are not meaningful as doses depend on the distribution of the radiopharmaceuticals. Thus, literature was reviewed and adjusted to topics as close as possible to the simulated data for CT and XFI.

There is a variety of influencing factors determining the exposure to the small animals. First of all, it is of great importance which radionuclide is used for which type of imaging procedure. Next it is relevant whether a pure nuclear medicine imaging procedure is used or whether a combined SPECT/CT or PET/CT investigation is performed and if the latter ones are done, whether the CT part is just intended for absorption and scatter correction or whether the CT part of the scan is also done to achieve anatomical information. The intended spatial and if relevant also the temporal resolution of the scan are very relevant parameters. Finally, a major aspect is, what kind of technology and especially what detector technology can be used for the scans.

It had been reported in various studies [[18], [19], [20], [21]] that CT doses for scans of living mice can be in the order of 50 mGy to 800 mGy for high resolution investigations. Other studies have been reporting approaches to reduce this exposure [[22], [23], [24]]. Also, there are approaches by companies to produce more efficient systems for CT scanning even in combined PET/CT systems as e.g., documented in [25]. This study documented dose values as low as 3 mGy for the scan of a mouse referring to short exposure times and optimized parameters for the X-ray source as well as suitable filters and an efficient detector setup. It should be mentioned, however, that this dose is corresponding to a spatial resolution of the scan of about 200 mm. This resolution is for sure sufficient for PET/CT investigations but cannot be compared to some of the micro-CT investigations cited before as there values for spatial resolution of 10 to 50 mm are used. This immediately shows that choosing the right parameters is relevant for dose efficient molecular imaging approaches and comparisons have to be made based on parameters for spatial and temporal resolution and required detail sizes etc. To conclude about the CT part of a PET/CT system for preclinical investigations it should be sufficient to use exposures of about 3 mGy in modern imaging setups. The same could hold for the CT part of SPECT/CT systems as long as it is estimated that a spatial resolution of 200 mm is sufficient for the SPECT/CT investigation as well. As SPECT imaging is not limited in spatial resolution by the positron range as PET imaging is, it might be possible that a higher spatial resolution could be of interest. However, currently this is not often implemented for other constraints and is also not meaningful taking into account the resolution achievable with XFI.

Regarding the nuclear medical imaging part itself the following considerations have to be made:

To calculate the dose to a target organ within the mouse, the following equation as introduced in the MIRD and standardized by Bolch et al [26]:

$$D\left(r_{T},T_{D}\right)=\sum_{r_{s}}\tilde{A}\left(r_{S},T_{D}\right)S\left(r_{T}\leftarrow r_{S}\right)$$

With the accumulated activities in the different source organs and the specific absorbed fractions S describing the energy transferred from the source organs to the target organs.

This indicates that two different types of information are necessary. On the one hand side it is mandatory to know the energy transfer radionuclide dependent from one organ to another. To evaluate this correctly, the physical parameters of the emitted particles need to be known, on the other hand the geometrical conditions between and in between the different organs need to be described to allow e.g., MonteCarlo calculations about the energy transfer. For animal imaging, animal models as described in [27,28] are used to perform such calculations. On the other hand, the accumulated activities over time in the different organs have to be determined. This is often done by building biokinetic models and determine the kinetic model parameters from experimental studies. As this is very time consuming, sometimes image-based studies using so called uptake values and time activity curves are used to estimate activities in the different source regions over time [29].

Based on such data exposures in SPECT and PET imaging for mice can be estimated. A standard protocol for preclinical PET imaging had been using 5 to 40 MBq of 18-FDG resulting to body doses of typically about 100 mGy (for 7 MBq injection) with peak doses in the bladder of up to 3 or 4 Gy. The detection efficiency can, however, be strongly increased by full field detection systems, improved detector material or configuration and improved reconstruction methods. In [25] a suitable image quality is reported for an injection of less than 1 MBq, which corresponds to a body dose of less than 15 mGy using the dose conversion coefficients introduced by [30].

For small animal SPECT imaging doses of between 60 and 900 mGy had been reported in 2004 [31] but also in the case of SPECT imaging a massive reduction of doses up to a factor 10 seems to be possible [32].

## Conclusion

Summing up, our findings highlight that X-ray fluorescence imaging proves to be a highly effective modality for achieving high sensitivity levels as shown in the example from in-vivo immune cell tracking with typical local amounts of just 200ng of iodine. In strong contrast, comparable detection levels using transmission imaging require 56 µg of iodine for single projection images and up to 123 µg for CT scans, depending on the resolution and clustering within the inhomogeneous model used. Given our objective to identify the lowest detectable amount in comparison to XFI, using the best-case scenario for transmission imaging is justifiable.

This remaining substantial difference further emphasizes XFI's superior sensitivity and its capability to distinguish between tissues containing the marker and bone. This capability is further enhanced in 3D reconstruction, where similar sensitivity to 2D XFI is maintained without an increase in dose. Consequently, XFI emerges as the preferred choice for in vivo applications involving low marker concentrations, particularly when tissue distributions are unknown. Our work shows in a quantitative way how to understand the large difference in sensitivity levels between XFI and X-ray absorption imaging when the same absorbed dose is used.

## CRediT authorship contribution statement

**Florian Grüner:** Writing – original draft, Methodology, Investigation, Formal analysis, Conceptualization. **Jan Scheunemann:** Writing – original draft, Investigation. **Christoph Hoeschen:** Writing – review & editing, Writing – original draft. **Thorsten Frenzel:** Investigation, Resources. **Theresa Staufer:** Writing – review & editing, Formal analysis.

## Declaration of competing interest

The authors declare that they have no known competing financial interests or personal relationships that could have appeared to influence the work reported in this paper.
