# Supplementary material for: Comparing the Achievable Sensitivity Limits of Synchrotron-based X-ray Fluorescence Imaging versus conventional X-ray absorption imaging and comparing absorbed dose levels including PET/SPECT
Source: Z Med Phys. 2025 Apr 25;36(2):212–25. doi: 10.1016/j.zemedi.2025.04.001 (PMC13316480; doi:10.1016/j.zemedi.2025.04.001)
Supplement: Supplementary Data 1 [file mmc1.docx]

**Supplementary Material**

**Flat Panel Detector Efficiency Calculation**

The efficiency curve for the flat panel detector was calculated using the cross-section for the photoelectric effect, based on the manufacturer’s information (Argonne National Laboratory. (2006). *Manual: PKIaSi XRD 0820 AN 120406*. Advanced Photon Source) that a 75 kVp spectrum would result in 56-58% efficiency. The crystal was assumed to be perfect and composed of 51.15% caesium, with the remainder being iodine (CsI). This makes a scintillator thickness of 200 μm very plausible, and given that this would result in cubic crystals, this depth was assumed. However, measurements by independent organisations often show lower efficiencies as they take into account also electronic noise and production deficiencies. That means the reported Detector Quantum Efficiencies (DQE)s in clinical or preclinical scenarios referring to complete detectors are often lower as they e.g., take into account electronic noise as well. Thus, the assumed real efficiency might be less resulting in that we overestimate the performance of the detector here and thus the efficiency that can be achieved by this scenario.

The detector efficiency was calculated assuming a 200 μm thick CsI scintillator crystal placed behind a 0.75 mm carbon fibre cover, with an assumed density of 2.2 g/cm³. In the Monte Carlo simulation, the resulting energies were multiplied by their respective efficiencies to obtain the total photon counts that a real detector would output, as a real panel detector would most likely not be energy-resolving.

**X-ray Transmission Calculations**

Based on water and different tissue compositions the transmissions for 53 keV photons (**Figure S1**) and the K_α_ lines of Iodine at 28.5 keV (**Figure S2**) were calculated.

| 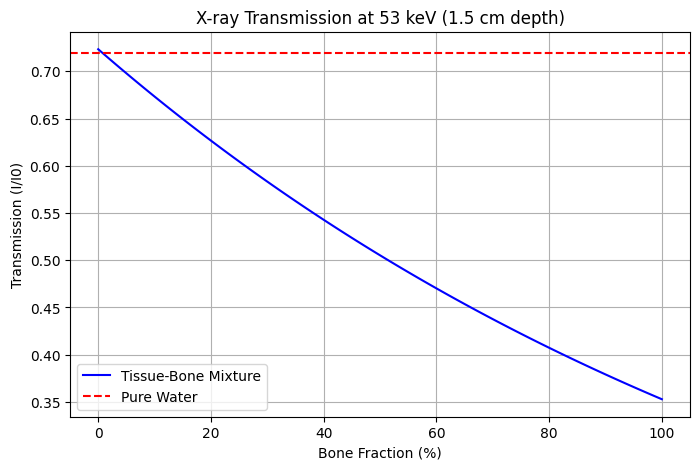  **Figure S1:** Transmission for photons through 1.5 cm of water or different bone tissue mixtures calculated for 53 keV. | 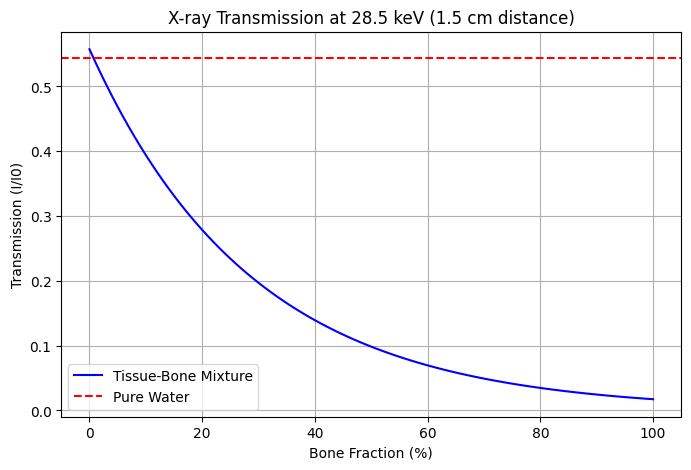  **Figure S2:** Transmission for photons through 1.5 cm of water or different bone tissue mixtures calculated using 28.5 keV. |
| --- | --- |

As expected, we can observe that at higher energies, the bone fraction has a smaller effect on the transmission. Consequently, in XFI, the outgoing attenuation is more dependent on the material composition than the incoming attenuation.

**Subtraction Imaging with Two Different Inhomogeneous Objects**

To illustrate that subtraction imaging is not feasible with two different inhomogeneous objects, we can demonstrate this without the need to create a new randomly generated second sphere or conduct a new transmission simulation. Instead, we can simply change the orientation of our existing sphere for one of the transmission images (**Figure S3**). This alteration creates a potential result for a new random distribution, which we can then be subtracted from the previously obtained image with the marker (**Figure S4).**

| 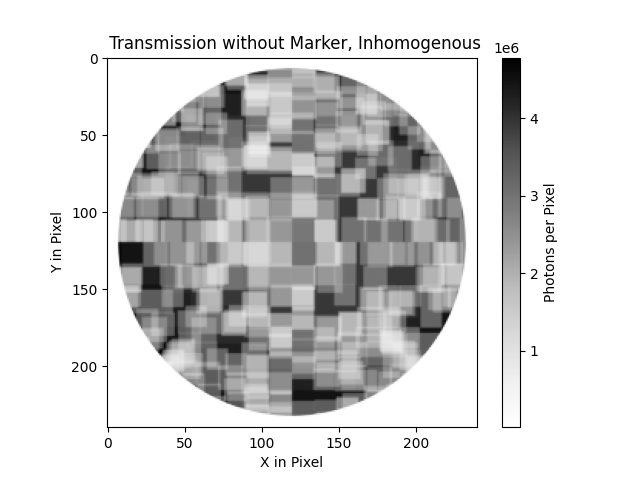  **Figure S3**: Transformation of Figure 9 by switching the x and y data to create a new random distribution of voxels, maintaining the same total amount of bone. | 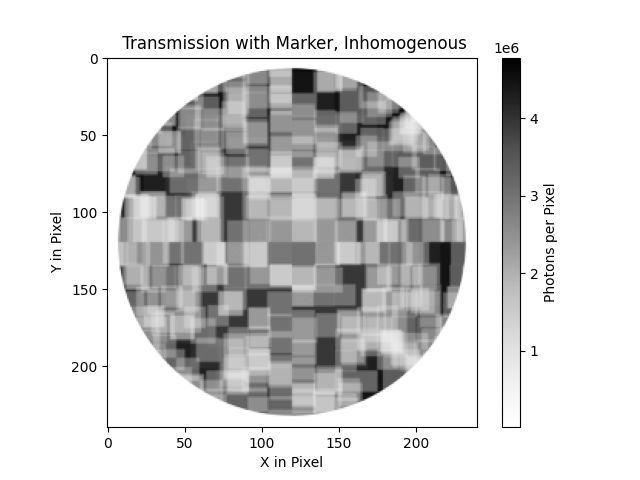  **Figure S4**: Identical to Figure 10, showing a transmission image of the inhomogeneous sphere. |
| --- | --- |
| 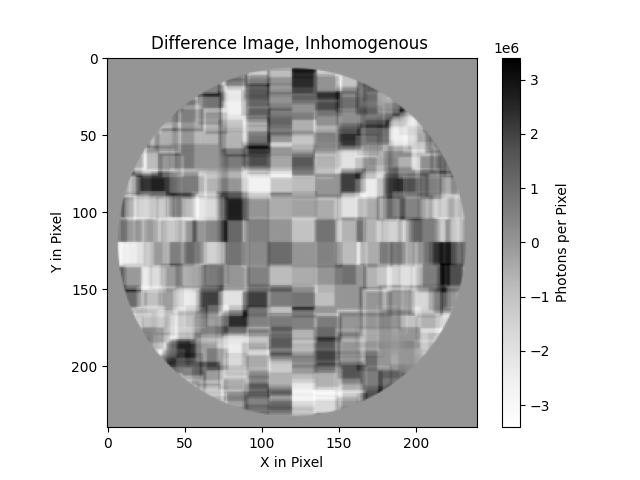  **Figure S5**: Subtraction image derived from Figure 15 and 16. In contrast to Figure 11, it does not display a clearly defined area of the marker, as the difference in attenuation caused by inhomogeneity is predominant. | 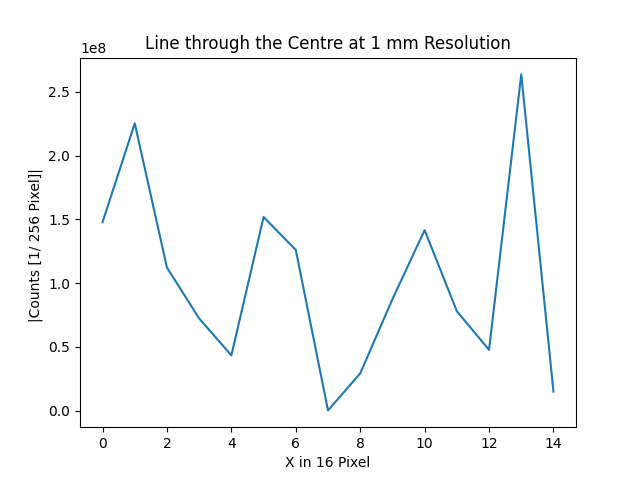  **Figure S6**: Absolute value of the sum over 1 mm pixel width along the centre line of the difference image of the inhomogeneous model. |

In the difference image (**Figure S5**), we observe the dominant effect of tissue inhomogeneity, which makes the location of the marker impossible to determine. At the centre, the difference between the two images decreases further, as shown in **Figure S6**. This is because there are more voxels along the path, leading to a more averaged representation of the bone. Consequently, the amount of iodine must be sufficiently high to differentiate tissue with the marker from bone. Once this threshold is reached, a single image is adequate, as will be demonstrated in the next chapter.

**XFI in 3D**

To demonstrate that XFI is capable of 3D reconstruction using an exposure dose identical to that of a 2D XFI reconstruction, we revisit the homogeneous model: a 30 mm diameter sphere of water containing 200 ng of iodine in a spherical marker solution. Our detector remains a single Amptek XR100 detector positioned at 150° to the incident beam.

In our X-ray Fluorescence Tomosynthesis (XFTS)^33^ approach we rotate the target similar to a CT scan; thus, our resolution is again determined by the size of our target divided by the number of projection angles.^[[1]](#footnote-1)^ We aim to compare the sensitivity for the same resolution as in the CT cases, which is 1 mm³. To avoid the overlapping of opposing beams, we utilize an uneven number of projection angles, so 31 instead of 30. Therefore, for each simulated pencil beam, we use 3.2e8 photons, ensuring that the sum of all incident photons remains identical to that in the 2D imaging cases.

Using back projection of the spectra from different angles and positions, each point in the 2D space of our scanning plane now possesses a summed spectrum from different angles. Back projection, rather than more advanced imaging modalities, is sufficient for this comparison, as the same approach was employed in our CT reconstruction.

At the centre of the sphere, we expect an identical spectrum for the XFTS case and the simulation of the spectrum from a 2D simulation. Towards the edges, where different Compton backgrounds from varying path lengths through the sphere and different outgoing transmissions accumulate, we anticipate larger differences in the overall shape of the spectra and their significance.

In this instance, our marker was placed at (X = 0 mm, Z = 8 mm). This position was chosen as a mean point between the centre and the edge of the sphere, to demonstrate that neither a perfectly symmetrical setup (marker at the centre) nor very low attenuation (marker at the edge) is necessary to reconstruct the marker position.

In these summed spectra, the amount of incident photons is identical to that of the spectra obtained from the previous single angle 2D scan. The total number of photons detected is reduced from 6.0e5 in the 2D-XFI case to 5.5e5 photons in the XFTS case. This is primarily due to a decrease in average path length of the incident beam through the sphere which in turn slightly decreases the Compton photon background in our signal region around 28.5 keV, as shown in **Figure S7**.


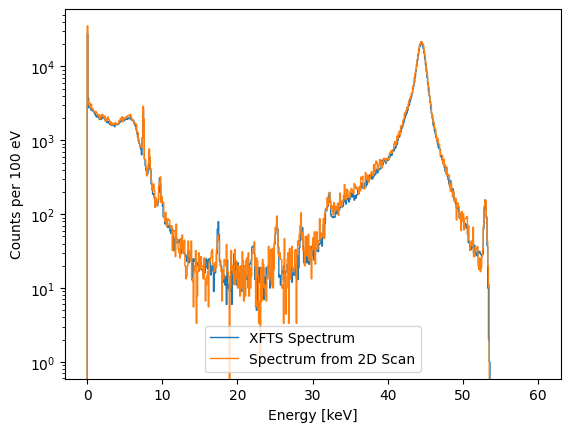


**Figure S7:** Comparison of the spectra of conventional XFI and the back-projected XFTS sum spectrum of (X= 0 mm, Z= 8 mm).

Subsequently, the signal and background of each pixel could be calculated through fitting of the fluorescence peak of the summed spectra. This would discriminate between background photons and fluorescence photons at the energy of the Kα-line of iodine in a measurement. In this case here, as we used a simulation, we can differentiate the types of photons (signal versus Compton) directly. If the corresponding significance is high enough (Z>3), we can also distinguish these photons with high precision in a measurement using fits.

Now we can examine the sinogram (**Figure S8**) that was used to create the spatial reconstruction. Since we have only one position with marker, we expect the sinogram to be sine-shaped. We can directly deduce the position of the marker based on the intersection with the y-axis at 0° and the amplitude; as anticipated, it is located at (0 mm, 8 mm). The negative sign of the sine wave is caused by the counterclockwise rotation of the target. Due to binning effects, the reconstruction via back projection shifts the marker position by 0.5 mm in X and minus Z direction in the Radon transformation image (**Figure S9)**.


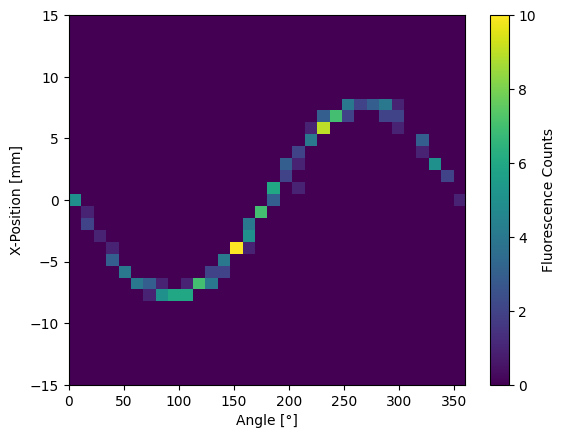


**Figure S8:** Sinogram of the XFTS reconstruction for the homogenous sphere at y = 0 mm.


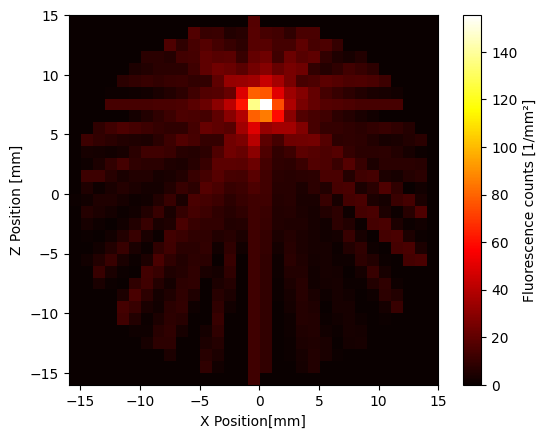


**Figure S9: Inverse** Radon transformation of the sinogram of the homogeneous water sphere created using XFTS and back projection.

The total number of fluorescence counts from the sinogram (165) is not identical to the number of photons projected onto our target pixel in the inverse radon transform (155). A higher resolution, achieved through more projection angles and a beam thinner than the marker volume, could improve this situation, allowing all measured photons to be precisely projected onto their origin. However, this is constrained by the requirement for the number of fluorescence counts per direction to be at least 1 on average. If we use additional ramp filters in the inverse Radon transformation, we can increase the localisation of the marker as shown in **Figure S10**:


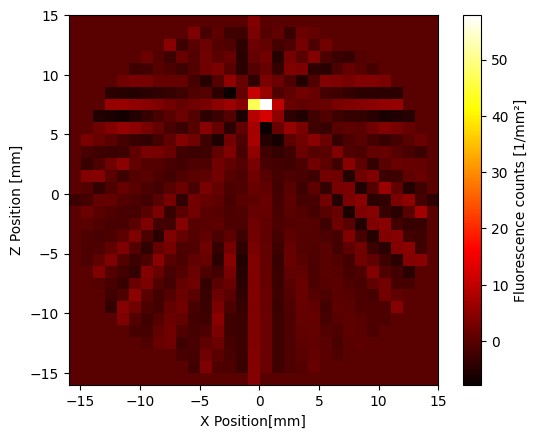


**Figure S10:** Inverse Radon Transformation of the sinogram of the homogeneous water sphere created using XFTS with ramp filters.

Note that, in contrast to a simulation, we would only be able to obtain such a sinogram indirectly during an actual measurement. Instead of employing an inverse Radon transformation, we would utilize the mathematically equivalent back projection of the spectra directly, as illustrated in **Figure S7**. This approach improves our signal-to-noise ratio before fitting the summed spectrum of a pixel, thereby omitting the need for a sinogram altogether. Hence, we use this back projection to calculate the significance:

$$Z_{XFTS}=\frac{132}{\sqrt{233}}=8.8$$

$$Z_{2D-XFI}=\frac{151}{\sqrt{273}}=9.1$$

We can see that in XFTS the significance (Z= 8.8) compared to a 2D XFI measurement (Z= 9.1) is, within statistical fluctuations, about the same. The somewhat reduced fluorescence counts in XFTS is due to fluorescence photons being projected onto adjacent voxels. We can calculate which number of photons we expect to be projected onto adjacent voxels. With 31 projection directions, the overlap between our 1mm² beam and a 1mm³ voxel is on average 80% at this marker position.^[[2]](#footnote-2)^ If we consider 80% of the total signal counts in the sinogram (165), we find that 132 is what we would expect.

If we take into account the signal and noise of all pixels from the sinogram that contain fluorescence photons we would obtain a higher significance of:

$$Z_{XFTS}(\mathrm{sinogram})=\frac{165}{\sqrt{327}}=9.1$$

The number of fluorescence counts in the sinogram is higher due to the lower average attenuation compared to the 2D scan: The average incoming path length decreases to about 1.39 cm, corresponding to 98.4% of the attenuation when we calculate all independent paths, while the average outgoing path length decreases to 1.4 cm, or 98.7% of the attenuation. As a result, the total transmission increases by 3%. Therefore, the increase in fluorescence counts from 151 ± 12 (in the 2D scenario) to 165 ± 13 (in the sinogram), representing a 9% increase, falls within the statistical expectation. The remaining difference arises from scattered photons that create fluorescence, which we can see in the sinogram when three pixels above each other all contain fluorescence counts.^[[3]](#footnote-3)^ This happened in 10% of the directions in our simulation.

The shorter average path length of the incoming beam through the entire sphere also explains the reduction in background photons.

The background of all pixels containing a signal is also higher than in the 2D case, but this is simply because the incident photons for all 51 pixels containing parts of the signal is higher than of just the 31 projections used in the back projection case. If we therefore use the total signal of the sinogram combined with the average background from 1e10 incident photons we obtain a maximum significance of:

$$Z_{max}= \frac{165}{\sqrt{233}}=10.8$$

This maximum could be achieved if a pencil beam from each direction perfectly hit a marker located 8 mm away from the centre of our sphere. The resolution is not limited by having significant measurements in every direction, but by the necessity of having a minimum fluorescence count in enough directions. Each direction that lacks fluorescence counts results in a further loss of resolution. If we observe the sinogram again, we see that each point has a very low number of fluorescence photons. On average, we have 5.3 photons per direction, with zero fluorescence photons in 3% of the directions. This reinforces the decision to use only 31 projection angles.

With this setup, we recorded 165 photons over 31 angles. With one direction yielding no fluorescence counts, the possible resolution worsens from 30/31 mm to 1 mm, which corresponds to a degradation in resolution of approximately 3%. A smaller amount of marker would therefore still deliver significant measurements but could harm spatial resolution. The measurement time for an XFTS measurement is greater than for a simple 2D measurement as it is mostly constrained by the speed of the sample holder motors and the number of movements scales with the number of projection angles.

Overall, a 3D reconstruction using XFTS, with significant results and 1 mm³ resolution, remains possible with 200 ng of marker without an increase in dose. This is remarkable, as such a reconstruction would not be feasible in conventional CT with the same dose. Notably, the transition from a 2D measurement to a 3D measurement occurs with minimal loss of significance while maintaining a constant dose.

1. This holds true because, with a 1 mm² beam, we have already fulfilled our resolution limit concerning the scan resolution. Therefore, our only limiting factor in achieving our 1 mm³ voxel resolution is the number of projections. [↑](#footnote-ref-1)
2. At the centre where all beams (with no displacement) overlap perfectly with our marker sphere, we achieve 100% of the signal projected onto the correct voxel. [↑](#footnote-ref-2)
3. It is impossible to shift a sphere with a 1 mm diameter on a 1 mm lattice to cover 3 cells in a line. [↑](#footnote-ref-3)
